# Does aligner refinement have the same efficiency in deep bite correction?: A retrospective study

**DOI:** 10.1186/s12903-024-04099-8

**Published:** 2024-03-15

**Authors:** Jessica Kang, Hyeran Helen Jeon, Nishat Shahabuddin

**Affiliations:** Department of Orthodontics, School of Dental Medicine, University of Pennsylvania, 240 South 40th Street, Philadelphia, PA 19104-6030 USA

**Keywords:** Deep bite, Invisalign, Clear aligners, Predictability, Refinement

## Abstract

**Background:**

Refinements are very common in clear aligner treatments. The aim of this study is to assess whether the predictability of deep overbite correction is similar over several refinements using clear aligners (Invisalign, Align Technology, San Jose, Calif) and examine the accuracy of vertical movement and inclination change of individual teeth.

**Methods:**

This retrospective study included 20 deep bite patients (7M and 13F; 32.63 ± 11.88 years old; an initial overbite of 5.09 ± 0.98 mm), consecutively treated from September 2016 and March 2023, who completed at least two sets of aligners, including refinements. The initial, predicted, and achieved models were exported from ClinCheck or OrthoCAD (Cadent Inc, Carlstadt, NJ) and superimposed via best-fit surface-based registration using SlicerCMF (version 4.9.0; cmf.slicer.org). We also examined 15 out of 20 patients who completed treatments. The overbite correction and changes in vertical movement and inclination for individual teeth were measured. Descriptive statistics and a paired t-test or Wilcoxon signed-rank test were performed. *P* < 0.05 was considered statistically significant.

**Results:**

The mean accuracy of overbite correction was 37.63% after 1^st^ set, followed by 11.19%, 6.32%, and 13.80% (2^nd^-4^th^ sets), respectively. There were statistically significant differences between the predicted and achieved vertical movements and inclination changes for all teeth for the 1^st^ and 2^nd^ sets. For the completed cases, the mean overbite correction was 38.54% compared to the initially planned overbite correction, which is similar to one of the 1^st^ set. Still, the vertical movements and inclination changes of all teeth present statistically significant differences between the initially planned and finally achieved movements except for maxillary lateral incisor torque.

**Conclusions:**

The most overbite correction occurs during the 1^st^ set of aligners, and refinement treatment does not significantly improve the deep bite correction.

**Supplementary Information:**

The online version contains supplementary material available at 10.1186/s12903-024-04099-8.

## Introduction

Deep bite is an excessive vertical overlap of the mandibular incisors by the maxillary incisors in maximum intercuspation, affecting approximately 15–20% of the US population [1–3]. Deep bite is correlated with sagittal molar relationship and is more frequent in the Class II population [4]. Treatment strategies for deep bites depend on incisor display and skeletal vertical patterns. Leveling a deep curve of Spee is one of the most common treatment strategies, including the intrusion of mandibular incisors, extrusion of posterior teeth, or a combination of both [5–7]. Mandibular incisor flaring is considered a relative intrusion. Depending on the gingival display or smile consonance, clinicians can intrude maxillary incisors [8, 9]. In hypodivergent patients, some extrusion of posterior teeth is beneficial to correct deep bite, while in hyperdivergent patients, it should be avoided. Instead, maxillary and mandibular incisor intrusion are preferred in hyperdivergent patients.

Clear aligners have become popular as an alternative to traditional braces [10]. Over 15 million patients have been treated with Invisalign as of March 2023. In recently published papers in 2020 and 2022, 25% of the annual treatment caseload was treated with clear aligners in the US [11] and Australia [12] and 93% of respondents provided the clear aligner treatment (CAT) in their practices [12]. The major factors in choosing CAT include case suitability, complexity, and patient cooperation [12]. Treatment predictability studies using CAT have been reported since 2009 [13–19]. Kravitz et al. found a 41% mean accuracy of CAT focused on the mesiodistal and labiolingual tip, vertical movement, and rotation of maxillary and mandibular anterior teeth [13]. Later, CAT studies categorized the treated cases into different movements and examined their specific efficiencies [14–20]. Still, deep bite correction using CAT is considered challenging with a 0.75–1.5 mm intrusion [21–23]. According to a recent survey in 2022, 62% of the respondents felt uncomfortable treating deep overbite using CAT, and 82% used CAT for deep bite correction sometimes/rarely/never [12]. In 2014 and 2021, Align Technology developed the Invisalign G5 and G8 to improve deep bite correction, including the pressure area, optimized attachments and bite ramps (G5), and individual activation on anterior teeth to improve leveling the curve of Spee and automatic placement of precision bite ramps for lower intrusion (G8). Despite many technological improvements, 50% of the average predictability of general tooth movements was reported in 2020 with 35% accuracy of mandibular incisor intrusion [24]. Most recently, we reported an average 33% predictability of overbite correction in deep bite patients after completing the first set of aligners [25]. We found a statistically significant difference between planned and achieved vertical movements and inclination changes after the initial set of aligner wear, indicating the necessary refinement treatment.

ClinCheck, a virtual treatment planning software designed for each Invisalign patient, enables orthodontists to visualize force systems and staging [24, 26]. However, treatment predictability is not 100% as we plan on the ClinCheck. Therefore, additional refinements and overcorrections are needed in almost every case [27–29]. The use of refinement aligners helps achieve closer to the final tooth position but requires more extended treatment time with patient cooperation. Kravitz et al. reported only 6% completed their CAT without any refinement, and 22.4% finished their CAT with one refinement [30]. On average, approximately 2–3 refinements and 22.8 months of treatment are expected for CAT patients. Given the recent 50% treatment accuracy following the use of the initial aligners, the current number of refinements appears to be justifiable [24]. However, the study to investigate whether additional refinement treatments provide similar treatment accuracy has yet to be done.

Our study aims to i) assess the accuracies of overbite correction over several refinements and ii) determine the discrepancy of individual teeth in vertical movements and inclination changes in the different sets of aligners. In addition, we investigated the fifteen completed deep bite cases for the accuracy of overall overbite correction and individual teeth movement change by comparing the initially planned and final tooth position.

## Materials and methods

This retrospective study was approved by the Institutional Review Board (IRB) at the University of Pennsylvania (Protocol #834,821), which is a follow-up study of Shahabuddin et al. [25]. This study examined 20 deep bite patients (7 M and 13 F; 32.63 ± 11.88 years old), who completed the initial and at least one refinement of Invisalign aligners. The initial aligners and subsequent refinements are called “sets” in this study. For example, the first set refers to the initial round of the upper and lower aligner sets, and the second set refers to the first refinements. All subjects were treated consecutively between September 2016 and March 2023, after the Invisalign G5 protocol for deep bite malocclusions and SmartTrack materials were developed. All patients were treated under the supervision of the experienced faculty in the Department of Orthodontics at the University of Pennsylvania School of Dental Medicine. Patients were instructed to wear aligners at least 22 h daily and change aligners every 1–2 weeks [31, 32]. The inclusion and exclusion criteria are similar to Shahabuddin et al. [25]: (1) All patients presented with dental deep bite malocclusion defined as ≥ 4 mm or ≥ 50% pretreatment overbite [19, 33–35], (2) non-growing patients (> 18 years old), (3) treatment in both arches with Invisalign, (4) initial, predicted, and achieved scans of first and second sets of aligners can be exported from the Invisalign Doctor website or OrthoCAD (Cadent Inc, Carlstadt, NJ), (5) completion of the first and second sets of aligners and (6) good compliance with consistent aligner wear. Exclusion criteria are defined as follows: (1) lack of completion of first and second sets of aligners, (2) poor compliance with the aligners, and (3) dental restorations before the refinement scan. 12 patients presented Angle Class I classification on molars, and 8 had Class II at least on one side. The cephalometric analysis was performed to assess the skeletal vertical pattern of patients using the Dolphin Imaging 3D software (version 11.9, Dolphin Imaging & Management Solutions, Chatsworth, CA, USA). Thirteen patients presented the normodivergent pattern with five hypodivergent and two hyperdivergent patterns based on SN-GoGn values.

**Fig. 1:**
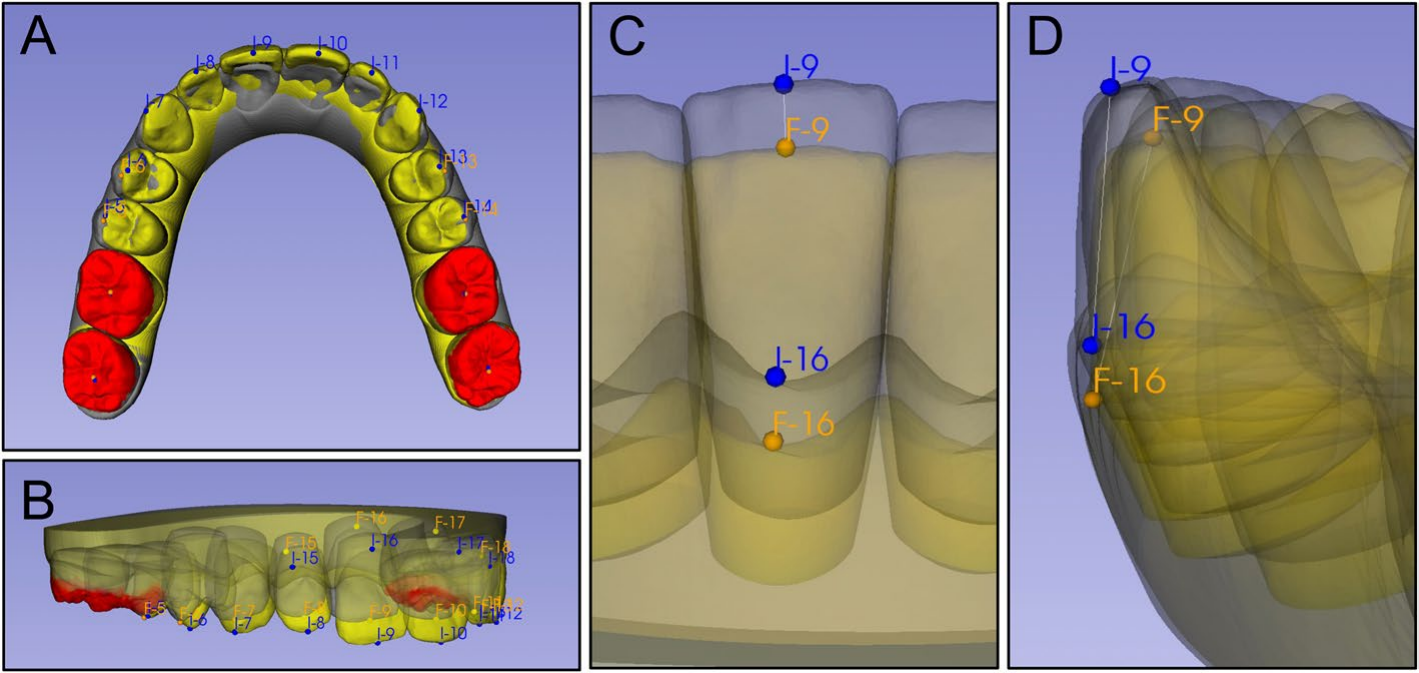
Superimposition and markers. The first and second molars, highlighted in red, served as the regions of interest for superimposition. Markers were placed from premolar to contralateral premolar cusp tips, centers of incisal edges, and at the intersections of the facial axis and gingival margin of each tooth. **A** and **B **Maxillary superimposition; **C** and **D **Vertical movement and inclination changes. **C** The difference in the vertical position of I-9 and F-9 represents the vertical movement of the tooth; **D** The difference in angulation from lines I-9 to I-16 and F-9 to F-16 represents the inclination change of the tooth

Overbite was measured as the largest distance between two opposing anterior incisors (central or lateral incisors) according to the American Board of Orthodontics grading system using the models on Clincheck [25, 36]. Previously, we compared the overbite measurements between ClinCheck and OrthoCAD, and found no statistically significant difference (*p* > 0.05) [25]. The initial, planned and achieved overbites for all aligner sets were measured. To further evaluate the vertical movement and inclination change of individual teeth, the initial, planned, and achieved models were exported as stereolithography files from ClinCheck, focused on 20 patients who completed the first and second sets of aligners. While ClinCheck may serve as a means to view the force system rather than a predictor of final tooth position, we use the terminology of “initial, predicted, and achieved” models in this study [24, 26, 37]. The initial and predicted models were exported from ClinCheck, while the achieved models were exported from either the initial model of the following set or directly exported from the final records in OrthoCAD if the patient completed their treatment. Four patients completed their treatment with two aligner sets and had final achieved models downloaded from OrthoCAD. The other sixteen patients had the final achieved models downloaded directly from ClinCheck. All initial, predicted and achieved models were deidentified and imported to analyze individual tooth movements using the 3D Slicer software via the SlicerCMF project (cmf.slicer.org, open-source, version 4.11.2). The model superimposition was carried out between i) initial and predicted and ii) initial and achieved models for the first and second sets separately using the best-fit surface registration focused on the occlusal surfaces of the first and second molar (Fig. 1A and B) [25, 38]. The average movements of maxillary and mandibular first and second molars were 0.05 ± 0.27 mm for the first set and 0.20 ± 0.21 mm for the second set. To assess the amount and direction of vertical movement and inclination changes, points were marked in the middle of the incisal edge (incisors), cusp tip (canines) and buccal cusp tip (premolars). Vertical change was measured at the mid-point of the incisal edges of anterior teeth and the cusp tips of canines and premolars (Fig. 1C). Inclination changes were examined for the maxillary and mandibular incisors. The points were placed on the middle of the incisal edge and the most apical point on the gingival margin along the long axis of the teeth (Fig. 1D). A line connecting two points was represented as the facial axis of the clinical crown (FACC). The inclination change was calculated as the difference between the two FACC lines between i) the initial and predicted or ii) initial and achieved models, demonstrating the change in the labiolingual position of the tooth. A positive value indicated that the change direction occurred in the same direction, while a negative value indicated the opposite direction. Per each set of the aligner, each patient had 40 vertical movements (20 for predicted and 20 for achieved) and 16 inclination changes (8 for predicted and 8 for achieved). A total of 383 teeth were measured for vertical change and 154 for inclination changes from the 20 patients for each aligner set. The contralateral teeth in the maxillary and mandibular arch were grouped together. The percent accuracy was measured as the (achieved amount/predicted amount) × 100%.

To further evaluate the final treatment outcomes, we followed up 15 patients (4 M and 11 F; 33.34 ± 12.71 years) who completed their treatment. We measured the overall overbite correction and the individual tooth movement, such as vertical movement and inclination changes, by comparing the initially planned tooth positions from the first ClinCheck and finally achieved tooth positions from debonding records. In total 287 teeth were measured for vertical change and 118 for inclination changes.

### Statistics

Using GraphPad Prism (version 8.4.0; San Diego, CA), descriptive statistics were used to calculate the mean and standard deviations. A paired t-test or Wilcoxon signed-rank test was performed to determine whether there were significant differences in overbite and tooth movements. For the interexaminer reliability, we compared our measurements of the first aligner set with the previous measurements by N.S [25]. To assess intraexaminer reliability, the same examiner (J.K.) remeasured the ten subjects at least four weeks after the initial measurements.* P* < 0.05 was considered statistically significant.

## Results

### Overbite correction of different sets of aligners

The average initial overbite for the 20 subjects was 5.09 mm ± 0.98 mm (Table 1). After the first set of aligners, we observed a 1.25 mm overbite improvement out of the 3.27 mm planned with 37.63% mean accuracy. After the second set of aligners, we found a 0.17 mm overbite improvement out of 2 mm planned with 11.19% mean accuracy, demonstrating a statistically significant difference compared to the accuracy of the first set (*p* < 0.05). 6.32% and 13.80% mean accuracies were observed after the third and fourth sets of aligners, respectively, while the sample size decreased on both sets of aligners due to the completed or ongoing treatment. All sets of aligners showed statistically significant differences between predicted and achieved overbite correction (*p* < 0.05). Due to the decrease in sample size, we focused on the first and second sets of aligners for an individual tooth movement analysis. An average of 29.30 ± 10.54 aligners were used for the first set, with an average treatment duration of 10.80 ± 4.36 months. For the second set of aligners, an average of 20.65 ± 11.79 aligners were used for 7.44 ± 4.06 months. We counted both upper and lower aligners as one. Initially, all 20 patients presented > 50% overbite, but 11 and 9 patients showed > 50% overbite after the first and second sets of aligners, respectively (Table 2).

**Table 1:** Overall overbite correction for deep bite patients of the present study (in mm)

Overbite Correction	N	Initial OB (mm)	Predicted (mm)		Achieved (mm)		Significance	Mean Accuracy (%)
			Mean	SD	Mean	SD		
1^st^ set	20	5.09	3.27	1.58	1.25	0.97	*	37.63
2^nd^ set	20	3.84	2.00	1.66	0.17	0.63	*	11.19*
3^rd^ set	11	4.30	2.49	1.91	0.21	0.38	*	6.32
4^th^ set	6	4.70	1.93	1.38	0.38	0.60	*	13.80

^*^Statistically significant at the *p* < .05 value

**Table 2:** Overbite correction for deep bite patients of the present study during 1st and 2nd aligner sets (in percentage)

OB percentage (%)	Initial OB	After 1^st^ set	After 2^nd^ set
< 50%	0	9	11
50% ≤ < 70%	8	6	5
70% ≤ < 100%	10	5	4
100% ≤	2	0	0
Total	20	20	20

For the first set, seven patients had the bite ramps, and all 20 patients had premolar attachments, which were optimized rotation, optimized deep bite, and conventional rectangular ones. The average overbite correction accuracy for seven patients with bite ramps was 48.41%, while one for those without was 31.83% (*p* > 0.05). In addition, 6 out of 20 patients used Class II intermaxillary elastics, including some patients with Class II canine and Class I molar relationships. The accuracy of OB correction in those with elastics was 53.90%, while those without elastics had 30.66% (*p* > 0.05). For the second set, only three patients had bite ramps, and 14 of the 20 patients had premolar attachments. The accuracy of overbite correction between those with bite ramps (22.52%) and those without bite ramps (9.19%) was not significant (*p* > 0.05). In addition, there was no significant difference between the 10 patients with Class 2 elastics (–1.55%) and those without elastics (23.93%) in the second set (*p* > 0.05). Lastly, there was no significant difference between the overbite correction accuracy in 14 patients with premolar attachments (5.00%) compared to 6 patients without premolar attachments (25.63%) (*p* > 0.05).

### Individual tooth movements of 1^st^ and 2^nd^ set of aligners

The predicted and achieved amount of vertical movement and inclination change for the first and second sets of aligners showed statistically significant differences for all teeth in maxillary and mandibular arches (*p* < 0.05) (Table 3). First, for the vertical measurements, all movements occurred in the same direction as planned in the first and second sets of aligners. In the maxillary arch, the first premolars had the highest mean accuracy of 48.72%, followed by the canines (45.61%), second premolars (42.51%), lateral incisors (25.09%), and central incisors (1.33%). In the mandibular arch, the canines demonstrated the highest mean accuracy of 45.64%, followed by the first premolars (44.75%), second premolars (44.45%), central incisors (42.13%), and lateral incisors (38.72%). For the inclination changes, the highest accuracy was observed in the mandibular central incisors (48.54%), followed by mandibular lateral incisors (35.41%), maxillary central incisors (26.69%), and maxillary lateral incisors (24.94%). The most significant discrepancy between the planned and achieved movement occurred in the vertical movement of the maxillary central incisors.

**Table 3:** Predicted and achieved changes in individual teeth movement during 1st and 2nd aligner sets

	1^st^ set							2^nd^ set						
	N	Predicted		Achieved		Significance	Difference	N	Predicted		Achieved		Significance	Difference
		Mean	SD	Mean	SD				Mean	SD	Mean	SD		
Maxillary Arch – Vertical (mm)								Maxillary Arch– Vertical (mm)						
U1 Vertical	38	1.09	0.91	0.01	1.07	*	1.08	38	0.91	0.89	0.08	0.71	*	0.84
U2 Vertical	38	1.11	0.93	0.28	0.92	*	0.83	38	0.77	0.73	0.09	0.66	*	0.69
U3 Vertical	38	1.10	0.76	0.50	0.76	*	0.60	38	0.75	0.52	0.29	0.52	*	0.45
U4 Vertical	38	0.66	0.43	0.32	0.53	*	0.34	38	0.40	0.30	0.14	0.50	*	0.26
U5 Vertical	36	0.52	0.34	0.22	0.33	*	0.30	36	0.36	0.22	0	0.46	*	0.36
Maxillary Arch – Inclination (°)								Maxillary Arch– Inclination (°)						
U1 Torque	38	6.51	5.52	1.74	5.97	*	4.77	38	4.36	3.96	0.68	2.27	*	3.68
U2 Torque	38	4.76	4.23	1.19	4.20	*	3.57	38	4.37	3.25	0.07	3.56	*	4.30
Mandibular Arch – Vertical (mm)								Mandibular Arch– Vertical (mm)						
L1 Vertical	40	2.71	2.02	1.14	1.56	*	1.57	40	2.26	2.44	0.61	0.63	*	1.65
L2 Vertical	37	2.41	1.83	0.93	1.17	*	1.48	37	2.29	2.96	0.58	0.81	*	1.93
L3 Vertical	40	1.83	1.63	0.84	0.97	*	1.00	40	1.68	2.12	0.40	0.63	*	1.28
L4 Vertical	40	0.74	0.68	0.33	0.50	*	0.41	40	0.83	1.38	0.18	0.33	*	0.66
L5 Vertical	38	0.37	0.41	0.16	0.43	*	0.20	38	0.50	0.74	0.12	0.27	*	0.38
Mandibular Arch – Inclination (°)								Mandibular Arch – Inclination (°)						
L1 Torque	40	7.40	5.84	3.59	4.91	*	3.81	40	5.21	5.29	0.71	3.22	*	4.50
L2 Torque	38	8.79	6.24	3.11	6.88	*	5.68	38	4.78	5.17	0.09	3.14	*	4.70

A negative sign indicates that the opposite movement was observed

^*^Statistically significant at the *p* < .05 value

Similarly, the second set of aligners showed statistically significant differences between the planned and achieved vertical movements and inclination changes (*p* < 0.05) (Table 3). All achieved vertical movements and inclination changes occurred in the same direction as planned for both arches. For the vertical measurements, in the maxillary arch the canines demonstrated the highest mean accuracy of 39.30%, followed by the first premolars (35.22%), lateral incisors (11.15%), central incisors (8.32%) and then second premolars (0.23%). In the mandibular arch, the second premolars showed the highest accuracy (36.11%), followed by the central incisors (26.89%), laterals (25.49%), canines (23.88%), and first premolars (21.13%). For the inclination changes, the mean accuracies were lower than the first set, with the highest accuracy in the maxillary central incisors (15.56%), followed by mandibular central incisors (13.54%), mandibular lateral incisors (1.78%), and maxillary lateral incisors (1.57%).

### Overbite correction for the completed cases

We further examined the 15 completed cases out of 20 patients. We compared the initially planned and final tooth positions to assess how much we achieved using multiple refinements. The initial mean overbite for the 15 patients was 5.07 ± 1.00 mm (Table 4). On average, 3.27 ± 1.10 sets of aligners with an average total of 68.13 ± 28.33 aligners were delivered to complete the treatments. The mean total treatment duration was 22.96 ± 12.34 months. We observed a 1.25 mm overbite improvement out of the 3.39 mm planned with 38.54% mean accuracy. The difference between the predicted and achieved overbite correction was statistically significant (*p* < 0.05).

**Table 4:** Overbite correction for completed deep bite patients of the present study

	N	Initial OB (mm)	Predicted (mm)		Achieved (mm)		Significance	Mean Accuracy (%)
			Mean	SD	Mean	SD		
Overbite Correction	15	5.07	3.39	1.65	1.25	0.87	*	38.54

^*^Statistically significant at the *p* < .05 value

### Individual tooth movements for the completed cases

The predicted and achieved vertical movement and inclination changes demonstrated the statistical significance for all teeth except maxillary lateral incisor torque (Table 5). Similar to the first and second sets of aligners, all vertical and inclination changes occurred in the same direction for the completed cases, except for vertical movements in the maxillary and mandibular arches occurred in the same direction as planned, except for the maxillary central incisors and maxillary canines. While 1.02 mm of intrusion was planned, 0.29 mm of extrusion occurred for the maxillary central incisors. 1.05 mm of intrusion was planned for the canines, and 0.11 mm of extrusion occurred. The predicted and achieved changes in the movements of maxillary central incisors and canines during 1st – 5th sets are assessed in Supplemental Table 1. In the 4th and 5th sets both maxillary central incisors and canines showed the opposite movement. Maxillary central incisors showed the most significant discrepancy between planned and achieved vertical movement.

**Table 5:** Initially predicted and finally achieved changes in individual teeth movement of the completed patients

	N	Predicted		Achieved		Significance	Difference		N	Predicted		Achieved		Significance	Difference
		Mean	SD	Mean	SD					Mean	SD	Mean	SD		
Maxillary Arch – Vertical (mm)									Mandibular Arch– Vertical (mm)						
U1 Vertical	30	1.02	0.96	-0.29	1.48	*	1.31	L1 Vertical	30	2.57	2.14	1.59	1.84	*	0.98
U2 Vertical	30	1.09	0.90	0.16	1.50	*	0.93	L2 Vertical	27	2.54	2.11	1.48	1.94	*	0.89
U3 Vertical	30	1.05	0.77	-0.11	1.18	*	1.16	L3 Vertical	30	1.66	1.67	1.02	1.76	*	0.63
U4 Vertical	30	0.61	0.39	0.07	0.76	*	0.54	L4 Vertical	28	0.63	0.59	0.56	1.50	*	0.07
U5 Vertical	28	0.47	0.28	0.18	0.46	*	0.29	L5 Vertical	28	0.35	0.34	0.09	1.61	*	0.26
Maxillary Arch – Inclination (°)									Mandibular Arch – Inclination (°)						
U1 Torque	30	6.59	5.87	2.50	5.24	*	4.08	L1 Torque	30	7.93	6.24	0.42	6.24	*	7.51
U2 Torque	30	4.07	4.48	2.08	7.35		2.62	L2 Torque	27	8.52	6.31	0.00	5.29	*	8.52

A negative sign indicates that the opposite movement was observed

^*^Statistically significant at the *p* < .05 value

### Intraexaminer and interexaminer reliability

The intraexaminer error between two times of measurements was determined using the intraclass correlation coefficient (ICC). The ICC ranged from 0.83—0.97, indicating good to excellent reliability. The interexaminer reliability was good to excellent, with the ICC ranging from 0.76—0.93.

## Discussion

Our findings demonstrated that the most significant overbite correction occurred after the first set of aligners (37.63%), and the accuracy of second aligner sets decreased by 70% (11.19%). The third set of aligners had 6.32% accuracy, with a decrease in sample size by almost half. Overbite correction showed a significant discrepancy i) between planned (3.27 mm) and achieved movements (1.25 mm) from the first set and ii) planned (2 mm) and achieved movements (0.17 mm) from the second set of aligners. In addition, the completed case analysis showed a large discrepancy between planned (3.39 mm) and achieved movements (1.25 mm) with 38.54% overbite correction accuracy, similar to one of the first set of aligners. A previous study reported a 1.5 mm bite opening with 40 completed deep bite patients using cephalometric analysis [19]. Henick et al. found 1.3 mm of mean overbite decrease and 0.65° of mean increase in mandibular plane angle (Sn-GoGn) in 24 skeletal deep overbite patients using the Invisalign appliance [1]. Recent systemic review papers in 2019 and 2020 demonstrated that deep bite correction using CAT ranges from 0.75–1.5 mm intrusion [21, 22]. The predictability of overbite reduction of completed deep bite cases was 30.5% in the group with an initial 4–7 mm overbite [39]. The ranges and accuracies of deep bite correction are similar to our findings, one of our first set and completed cases, demonstrating that major overbite correction occurs in the first set of aligners and refinement treatment does not significantly improve the overbite correction. A possible reason for a decrease in the accuracy of overbite correction during refinements might be a continuous posterior bite block effect. Even though we plan for posterior extrusion in the ClinCheck setup, an occlusal covering in aligners combined with mastication forces might counteract their movements. In addition, the overall overbite correction with refinements is not linear, reaching the plateau.

A significant discrepancy was found between planned and achieved movements in the first and second sets of aligners, even in the completed cases. Maxillary central incisors have the lowest treatment accuracy of intrusion in the first set of aligners, with an average of 1.33%, similar to the previous studies [17, 25, 38]. We planned an average of 1.09 mm and 0.91 mm intrusion on maxillary central incisors in the first and second sets, respectively. However, we found 0.01 mm and 0.08 mm of intrusion from the superimposition of initial and achieved models. Previous studies reported a large discrepancy in the vertical movement in maxillary central incisors, even the opposite movement, using a best-fit surface-based registration for the superimposition [17, 38]. The opposite movement might be, in part, an outcome of the superimposition method, as previously described [38]. The potential bite block effect of CAT could contribute to the unexpected molar intrusion, resulting in the appearance of incisor extrusion on the posttreatment models after superimposition.

In our study with the completed cases, we found an average of 3.27 ± 1.10 sets of aligners were delivered with a total number of 68.13 ± 28.33 aligners. The average treatment duration was 22.96 months ± 12.34 months. These findings are similar to other studies. A recent survey reported that 81% of annual CAT caseloads require refinements with an average of 2.02 ± 1.76 per patient to complete the treatments, including different malocclusions [12]. Kravitz et al. demonstrated that Invisalign treatment required an average of 2.5 refinement scans with an average of 64.1 aligners [30]. Only 6% of patients could finish their treatment without any refinement. The average length of Invisalign treatment was 22.8 months. Interestingly, 17% of patients switched from Invisalign to fixed braces during the treatment.

The comparison of treatment outcomes in severe deep bite patients with > 5 mm and > 60% overbite between Invisalign and fixed appliance was carried out using the cephalometric analysis and peer assessment rating [40]. A significant difference in nasion-menton (mm) and mandibular plane-mandibular first molar (mm) was observed between Invisalign and fixed appliance groups, whereas there were no significant differences in the peer assessment rating analysis or total treatment duration: Invisalign (31.9 months) vs. fixed appliance (32.7 months). Therefore, the authors preferred using Invisalign over conventional fixed appliances in hyperdivergent patients with a deep overbite. Rozzi et al. compared the leveling of the COS between Invisalign and fixed appliances [7]. While the fixed appliance group showed a significant posterior teeth extrusion with mandibular incisors flaring, the Invisalign group demonstrated a significant mandibular incisors intrusion with excellent control in the incisors proclination. Henick et al. compared the skeletal deep bite correction between Invisalign and fixed appliances [1]. The mean overbite reduction was 1.3 mm and 2.0 mm for the Invisalign and fixed appliance groups, respectively. The mean increase in mandibular plane angle was 0.65° and 1.15° for the Invisalign and full fixed appliance groups, respectively. Based on previous studies, Invisalign and fixed appliance showed a similar amount of lower incisor intrusion and treatment duration, whereas posterior extrusion is superior with fixed appliance [24, 41, 42].

Leveling the mandibular curve of Spee (COS) is a common mechanism for deep bite correction. Goh et al. examined the 42 subjects treated with Invisalign aligners without auxiliaries such as intermaxillary elastics and bite ramps from 2013 to 2019 and reported a mean 35% accuracy of COS leveling [6]. Therefore, overcorrection in the leveling of mandibular COS is recommended, focusing more on the extrusion of mandibular first molars. Clinicians can exaggerate the reverse COS in the ClinCheck setup, leading to an anterior open bite with heavy posterior occlusal contacts [27]. In addition, leveling the curve of Wilson can improve a deep bite correction with the mandibular posterior extrusion through 5° of the buccal crown tip [27].

We can also consider using auxiliaries such as bite ramps and Class II elastics. Bite ramps are commonly used in brachycephalic patients and are automatically added if the prescribed lower incisor intrusion is more than 1.5 mm. The main effect of bite ramps is to avoid the bite block effect and allow room for posterior extrusion. The impact on upper incisors from intrusion seems to be minimal, given that we typically maintain a disoccluded jaw position with the freeway space. The effects of bite ramps have been examined in several studies. Blundell et al. [43] and Brenner [44] reported no or minimal effect of bite ramps. The effect of different locations of bite ramps is not statistically significant whether it is located on the upper six incisors versus the upper four incisors [1]. Class II elastics can enhance mandibular posterior extrusion and mandibular incisor proclination. In cases where the maxillary canine intrusion is planned, relocating the precision hook to the first premolar can be implemented to mitigate potential conflicts in the force system. In addition, overtreatment is commonly recommended in the final ClinCheck setup to overcome the low accuracy of deep bite correction using CAT, including an anterior edge-to-edge or slight open bite, heavy posterior occlusal contacts, a reverse COS in the mandibular arch [45]. Voudouris et al. suggested 150–200% overbite supercorrection depending on the initial severity of overbite, the interproximal reduction during intrusion, and the use of chewies between the maxillary and mandibular anterior teeth [35]. Dome-shaped attachments on premolars (G5) and molars (G7) and horizontal attachments can be used on mandibular posterior teeth for the anchorage setup for the anterior intrusion [37, 46]. Interestingly, a recent study discovered no notable difference in the effectiveness of deep bite correction with aligners when comparing optimized attachments to conventional ones [47].

A hybrid aligner treatment can be beneficial for deep bite correction, using CAT in the maxilla and a fixed appliance in the mandible to enhance mandibular anterior intrusion and posterior extrusion [24, 48]. In the 2022 survey, 47% of respondents combined CAT with fixed appliances as part of the initial treatment plan [12]. Moreover, the staggered lower anterior intrusion can be considered, comprising an alternate intrusion of canines and incisors [4].

Although the palatal rugae are acknowledged as a stable reference for the maxillary arch, there remains a lack of consensus regarding a stable reference for the mandibular arch [49, 50]. Previous studies support that the mucogingival junction and mandibular tori in adult patients could be accurate and reliable references for mandibular models. Conversely, the buccal and lingual alveolar surfaces near the dentition without mandibular tori are deemed inappropriate [51, 52]. As ClinCheck only presents the crowns and gingiva, we cannot use those structures for superimposition. Instead, superimposition on the occlusal plane, stable teeth, and best-fit algorithms have been used in previous studies [17, 32, 38, 53]. Utilizing an occlusal plane for superimposition becomes viable when reference points, such as the molars, undergo minimal displacement [38, 53]. Furthermore, employing computer software for superimposition through a best-fit algorithm, instead of manual registration, can diminish operator errors by minimizing the reliance on user-selected landmarks [54, 55].

We have some limitations in our study. First, a lack of stable landmarks for the ClinCheck superimpositions is one main limitation as described above [25]. Second, 65% of our patients present the normodivergent pattern with 25% hypodivergent and 10% hyperdivergent patterns. Generally, skeletal deep bite correction is more challenging than dental deep bite correction [3, 56]. Third, our study has a small sample size, which restricts the scope of our interpretations. This study serves as a follow-up to a previously published study [25], and we encountered some loss of samples from the initial cohort during the follow-up period. A larger number of samples can provide a more comprehensive understanding of deep bite correction using CAT in addition to the effects of the auxiliaries.

## Conclusions

Our study demonstrates that most deep bite correction occurs in the first set of aligners, and additional refinement treatment does not significantly improve the deep bite correction. Still, the discrepancy between planned and achieved movements in individual teeth exists in refinements. Even after overcorrection and several refinements, complete deep bite correction is challenging with CAT. To improve the low predictability of deep bite correction, hybrid treatments are recommended, using CAT in the maxillary arch and fixed appliance in the mandibular arch.

### Supplementary Information

**Additional file 1: Supplemental Table 1.** Predicted and achieved changes in the movements of maxillary central incisors and canines during 1st – 5th aligner sets.

## Data Availability

The datasets used and/or analyzed during the current study are available from the corresponding author upon reasonable request.
