# Supplementary material for: Does aligner refinement have the same efficiency in deep bite correction?: A retrospective study
Source: BMC Oral Health. 2024 Mar 15;24:338. doi: 10.1186/s12903-024-04099-8 (PMC10943900; doi:10.1186/s12903-024-04099-8)
Supplement: Supplementary file 1 — Additional file 1: Supplemental Table 1. Predicted and achieved changes in the movements of maxillary central incisors and canines during 1st – 5th aligner sets. [file 12903_2024_4099_MOESM1_ESM.docx]

|  |  | N | Predicted | | Achieved | | Significance | Difference |
| --- | --- | --- | --- | --- | --- | --- | --- | --- |
|  |  |  | Mean | SD | Mean | SD |  |  |
| 1^st^ | U1 Vertical | 38 | 1.09 | 0.91 | 0.01 | 1.07 | * | 1.08 |
|  | U3 Vertical | 38 | 1.10 | 0.76 | 0.50 | 0.76 | * | 0.60 |
| 2^nd^ | U1 Vertical | 38 | 0.91 | 0.89 | 0.08 | 0.71 | * | 0.84 |
|  | U3 Vertical | 38 | 0.75 | 0.52 | 0.29 | 0.52 | * | 0.45 |
| 3^rd^ | U1 Vertical | 22 | 1.01 | 0.83 | 0.45 | 0.61 | * | 0.56 |
|  | U3 Vertical | 22 | 0.67 | 0.58 | 0.08 | 0.48 | * | 0.59 |
| 4^th^ | U1 Vertical | 10 | 1.40 | 0.88 | -0.13 | 0.96 | * | 1.52 |
|  | U3 Vertical | 10 | 0.65 | 0.67 | -0.08 | 0.50 | * | 0.73 |
| 5^th^ | U1 Vertical | 6 | 0.90 | 0.73 | -0.41 | 0.34 | * | 1.31 |
|  | U3 Vertical | 6 | 0.58 | 0.53 | -0.27 | 0.30 | * | 0.85 |

**Supplemental Table 1.** Predicted and achieved changes in the movements of maxillary central incisors and canines during 1^st^ – 5^th^ aligner sets
